# Pretreatment gut microbiome predicts chemotherapy-related bloodstream infection

**DOI:** 10.1186/s13073-016-0301-4

**Published:** 2016-04-28

**Authors:** Emmanuel Montassier, Gabriel A. Al-Ghalith, Tonya Ward, Stephane Corvec, Thomas Gastinne, Gilles Potel, Phillipe Moreau, Marie France de la Cochetiere, Eric Batard, Dan Knights

**Affiliations:** Université de Nantes, EA 3826 Thérapeutiques cliniques et expérimentales des infections. Faculté de médecine, 1 Rue G Veil, Nantes, 44000 France; Department of Computer Science and Engineering, University of Minnesota, Minneapolis, MN 55455 USA; Biomedical Informatics and Computational Biology, University of Minnesota, Minneapolis, MN 55455 USA; Biotechnology Institute, University of Minnesota, St. Paul, MN 55108 USA; Nantes University Hospital, Microbiology Laboratory, Nantes, France; Hematology Department, Nantes University Hospital, Nantes, France

**Keywords:** Bloodstream infection, Chemotherapy, Intestinal microbiome, Prediction

## Abstract

**Background:**

Bacteremia, or bloodstream infection (BSI), is a leading cause of death among patients with certain types of cancer. A previous study reported that intestinal domination, defined as occupation of at least 30 % of the microbiota by a single bacterial taxon, is associated with BSI in patients undergoing allo-HSCT. However, the impact of the intestinal microbiome before treatment initiation on the risk of subsequent BSI remains unclear. Our objective was to characterize the fecal microbiome collected before treatment to identify microbes that predict the risk of BSI.

**Methods:**

We sampled 28 patients with non-Hodgkin lymphoma undergoing allogeneic hematopoietic stem cell transplantation (HSCT) prior to administration of chemotherapy and characterized 16S ribosomal RNA genes using high-throughput DNA sequencing. We quantified bacterial taxa and used techniques from machine learning to identify microbial biomarkers that predicted subsequent BSI.

**Results:**

We found that patients who developed subsequent BSI exhibited decreased overall diversity and decreased abundance of taxa including Barnesiellaceae, Coriobacteriaceae, *Faecalibacterium*, *Christensenella*, *Dehalobacterium*, *Desulfovibrio*, and *Sutterella*. Using machine-learning methods, we developed a BSI risk index capable of predicting BSI incidence with a sensitivity of 90 % at a specificity of 90 % based only on the pretreatment fecal microbiome.

**Conclusions:**

These results suggest that the gut microbiota can identify high-risk patients before HSCT and that manipulation of the gut microbiota for prevention of BSI in high-risk patients may be a useful direction for future research. This approach may inspire the development of similar microbiome-based diagnostic and prognostic models in other diseases.

**Electronic supplementary material:**

The online version of this article (doi:10.1186/s13073-016-0301-4) contains supplementary material, which is available to authorized users.

## Background

Hematopoietic stem cell transplantation (HSCT) is commonly applied as curative treatment in patients with hematological malignancy [1]. A frequent side effect of myeloablative doses of chemotherapy used during the HSCT procedure is gastro-intestinal (GI) mucositis [2].

A recent model, introduced by Sonis, described a process for bacterial infection due to GI mucositis [3]. It includes an ulcerative phase with increased permeability and damage to the intestinal mucosal barrier. This promotes bacterial translocation, defined as the passage of bacteria from the GI tract to extra-intestinal sites, such as the bloodstream [4]. Bacteremia, or bloodstream infection (BSI), remains a common life-threatening complication with well-documented morbidity and mortality in patients with cancer [5]. In a recent study, the overall rate was 9.1 BSIs per 1000 patient-days with a 28-day case mortality rate of 10 % and 34 % in case of *P. aeruginosa*. [6]. Another study reported that the overall incidence of BSI was 7.48 episodes per 1000 hospital stays for neutropenic hematological patients, with 11 % of the patients requiring intensive care unit admission and resulting in an overall case-fatality rate at 30 days of 12 % [7]. Furthermore, BSI is particularly frequent during the early transplant period due to the intensive chemotherapy regimen administered prior to HSCT [8], but there is currently no way to predict or prevent it.

While the model of pathobiology of mucositis reported above is silent on the role of the intestinal microbiome, Van Vliet et al. proposed a potential role for the intestinal microbiome in BSI [9]. A previous study reported that intestinal domination, defined as occupation of at least 30 % of the microbiota by a single bacterial taxon, is associated with BSI in patients undergoing allo-HSCT [10].

However, the impact of the intestinal microbiome before treatment initiation on the risk of subsequent BSI remains poorly studied. We hypothesized that patients who entered the hospital with a diverse microbiome dominated by operational taxonomic units (OTUs) that were previously associated with gut homeostasis would be less likely to acquire a BSI. Thus, the objective of our work was to use fecal samples collected prior to chemotherapy to identify biomarkers in the fecal microbiome that predict the risk of subsequent BSI.

## Methods

### Study patients and fecal sample collection

Participants with non-Hodgkin lymphoma (NHL) were recruited in the hematology department of Nantes University Hospital, France, as reported in our previous study [11]. Briefly, in this study, we excluded patients with a history of inflammatory bowel diseases, those exposed to probiotics, prebiotics, or broad-spectrum antibiotics, and those administered nasal-tube feeding or parenteral nutrition in the month prior to initiation of the study. Participants received the same myeloablative conditioning regimen for 5 consecutive days, including high-dose Carmustine (Bis-chloroethylnitrosourea), Etoposide, Aracytine, and Melphalan, and allogeneic HSCT occurred on the seventh day. Most of the participants received antibiotic prophylaxis before the conditioning therapy based on penicillin V and/or cotrimoxazole, which was stopped on the day of the hospital inpatient admission. Therefore, no patient had ongoing antibiotic treatment at the time of the sample collection and all the patients stopped the antibiotic treatment on the same day: hospital inpatient admission (Day 0).

BSI, the endpoint of the study, was assessed during inpatient HSCT hospitalization, following standard Centers for Disease Control and Prevention definitions of a laboratory-confirmed bloodstream infection. We collected a fecal sample from all participants. The fecal sample was collected on hospital inpatient admission (Day 0), prior to administration of the high-dose chemotherapy conditioning the transplant, and was stored at −80 °C until analysis.

### DNA extraction, PCR amplification of V5-V6 region of bacterial 16S ribosomal RNA genes, and pyrosequencing

The genomic DNA extraction procedure was based on the QIAamp® DNA Stool Minikit (Qiagen, Hilden, Germany), as reported in our previous study [11]*.* Then, for each sample, we amplified 16S ribosomal RNA (rRNA) genes, using a primer set corresponding to primers 784 F (AGGATTAGATACCCTGGTA) and 1061R (CRRCACGAGCTGACGAC), targeting the V5 and V6 hypervariable 16S rRNA gene region (~280 nt region of the 16S rRNA gene) [12]. Pyrosequencing was carried out using primer A on a 454 Life Sciences Genome Sequencer FLX instrument (454 Life Sciences-Roche, Brandford, CT, USA) with titanium chemistry at DNAVision (Charleroi, Belgium).

### Sequence analysis

The 16S rRNA raw sequences were analyzed with the QIIME 1.8.0 software [13]. Sequences were assigned to 97 % ID OTUs by comparing them to the Greengenes reference database 13_8 [14]. We represented beta diversity, based on Unweighted UniFrac distances, with principal coordinate analysis (PCoA). We applied the PERMANOVA method on the previously obtained dissimilarity matrices to determine whether communities differ significantly between fecal samples of patients who ultimately did or did not develop BSI. PERMANOVA was performed using 1000 permutations to estimate *p* values for differences among patients with different BSI status. We computed alpha diversity metrics, using both non-phylogeny and phylogeny-based metrics, and tested differences in alpha diversity with a Monte Carlo permuted t-test. We performed a non-parametric t-test with 1000 permutations to calculate the *p* values for differences among patients with different BSI status. We used PICRUSt, a computational approach to predict the functional composition of a metagenome using marker gene data (in this case the 16S rRNA gene) and a database of reference genomes [15].

### Statistical analysis

We developed a BSI risk index corresponding to the difference between a patient’s total relative abundance of taxa associated with protection from BSI and the patient’s total relative abundance of taxa associated with development of a subsequent BSI. In detail, we included in the BSI risk index all the taxa with a false discovery rate (FDR)-corrected *p* value less than 0.15. FDR was applied at each taxonomy level separately. For the predictive panel, the primary assessment of the relevance of the taxa is the accuracy of the predictions rather than the significance of the individual features, although the FDR threshold used still has the standard interpretation for statistical significance. The BSI risk was calculated using the sum of relative abundances of the taxa that were significantly associated with BSI minus the sum of the relative abundances of the taxa that were associated with protection from BSI (Additional file 1). Importantly, we assessed the accuracy of predictions by predicting the risk index for a given patient using predictive taxa identified using only other patients, in order to avoid information leak. The leave-one-out procedure consisted of holding a single patient out from the entire analysis at each iteration, in which the held-out sample represented a novel patient from the same population. This assessed the ability of the classifier to predict BSI risk for one patient based on their pre-chemotherapy microbiome, using a model trained only on the pre-chemotherapy microbiomes of other patients. We then retrained the model one last time on the entire dataset to report the taxa included in the predictive panel. To assess variability in the predictive strength of the model depending on training data selection, we plotted receiver-operating characteristic (ROC) curves and computed the area under the curve (AUC) values on ten sets of predictions obtained from tenfold cross-validation using ROCR package in R. In parallel to the BSI risk index analysis, we also performed Random Forest (RF) classification with 500 trees and tenfold cross-validation [16].

To determine whether differences in sequencing depth across samples could be a confounding factor in our estimates of diversity, we compared sequencing depths between BSI and non-BSI patients using a Mann–Whitney U test. To evaluate the effects of different sequencing depth across samples on diversity estimates resulting from OTU picking [17], we subsampled the original sequencing data to an even depth of 3000 sequences per sample prior to picking OTUs. We then re-calculated alpha diversity (observed species, phylogenetic diversity) and performed a Mann–Whitney U test to compare alpha diversity between BSI and control participants. We repeated this subsampling procedure at 2000 and 1000 sequences per sample.

## Results

### Patient and fecal sample characteristics

The study included 28 patients with NHL undergoing allogeneic HSCT. Of the fecal samples collected, a total of 280,416 high-quality 16S rRNA-encoding sequences were identified, representing 3857 OTUs. Since samples contained between 3041 and 26,122 sequences, diversity analyses were rarefied at 3041 sequences per sample (Additional file 2). We identified the reported taxon associations using non-rarefied data normalized to relative abundances.

BSI was reported in 11 patients (39 % [24–58 %]), at a mean ± standard deviation of 12 ± 1 days after sample collection. Two patients (18.2 % [5.1–47.7 %]) developed *Enterococcus* BSI, four (36.4 % [15.0–64.8 %]) developed *Escherichia coli* BSI, and five (45.5 % [21.3– 72.0 %]) patients developed other Gammaproteobacteria BSI. Here and henceforth, qualitative data are reported as percentage [95 % confidence interval] and quantitative data are reported as medians [25–75 % percentile] unless otherwise noted. As detailed in Table 1, antibiotic prophylaxis based on penicillin V and/or cotrimoxazole was received before admission in nine (82 %, 52–95) BSI patients and 15 (88 %, 65–97) patients without BSI (Fisher’s exact test, two-sided *p* value = 0.99). Importantly, antibiotic prophylaxis was not associated with a specific microbiome composition (Additional file 3). Moreover, all the patients received chemotherapy and broad spectrum antibiotics before the HSCT hospitalization, by a median delay of 4 months.

**Table 1:** Characteristics of the study population

	BSI group (*n* = 11)	No BSI group (*n* = 17)	*p* value
Age (years)	59 [46–61]	54.5 [45–60]	0.80
Sex (male)	7 (64 %, 35–85)	13 (76 %, 53–90)	0.75
Body mass index	24 [22–28]	25 [24–28]	0.90
Antibiotic prophylaxis	9 (82 %, 52–95)	15 (88 %, 65–97)	0.99
Penicillin V	8 (72 %, 49–92)	6 (35 %, 17–59)	0.12
Cotrimoxazole	7 (63 %, 36–85)	12 (70 %, 47–87)	0.99
ICU admission	1 (9.0 %, 1.6–37.7)	2 (11.8 %, 2.0–37.8)	0.99
Days of neutropenia	9.0 [8.5-10.0]	10 [9–11]	0.27
Previous chemotherapy (months)	4.0 [3–7.5]	4 [3–5]	0.44
Previous antibiotic treatment (months)	4.0 [2–5]	4 [3–5]	0.66
Other comorbidities, hypertension	4 (36.4 % 12.7–68.4)	2 (11.8 %, 2.0–37.8)	0.28
Diffuse large B-cell lymphoma	9 (81.8 %, 47.8–96.8)	10 (58.9 %, 36.0–78.4)	0.39
Follicular lymphoma	0 (0.0 %, 0.0–25.8)	2 (11.8 %, 2.0–37.8)	0.67
Burkitt lymphoma	0 (0.0 %, 0.0–25.8)	1 (5.9 %, 1.0–27.0)	0.99
Mantle cell lymphoma	2 (57.1 %, 25.0–84.2)	3 (17.6 %, 6.2–41.0)	0.39
Anaplastic large cell lymphoma	0 (0.0 %, 0.0–25.8)	1 (5.9 %, 1.0–27.0)	0.99

BSI, Bloodstream infection; ICU, Intensive Care Unit; NHL, non-Hodgkin lymphoma

Quantitative data are shown as median [1st and 3rd quartile]; fractional data are shown as mean [lower-upper bounds of 95 % confidence interval]

### Decreased diversity in pre-chemotherapy fecal samples associated with subsequent BSI

**Fig. 1:**
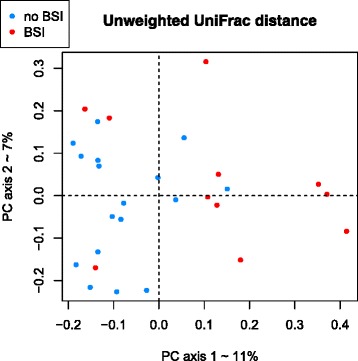
Beta-diversity comparisons of the gut microbiomes of fecal samples from samples collected prior to treatment in patients who developed subsequent BSI (*n* = 11) and in patients who did not develop subsequent BSI (*n* = 17). The first three axes are shown of principal coordinate analysis (PCoA) of Unweighted UniFrac distances between patient bacterial communities. The proportion of variance explained by each principal coordinate axis is denoted in the corresponding axis label. *The plot* shows a significant separation between fecal samples from patients who developed subsequent BSI and in patients who did not develop subsequent BSI (PERMANOVA, *p* = 0.01)

PCoA of fecal samples collected prior to treatment, based on 16S rRNA sequences of unweighted UniFrac distance metric, showed differences between fecal samples of patients who did or did not develop BSI (PERMANOVA, two-sided *p* value = 0.01) (Fig. 1). Differences were not significant when using weighted UniFrac. In our previously published studies we have found consistently that at the level of OTUs, unweighted UniFrac provides better power than weighted UniFrac for discriminating experimental groups. We also used a standard machine-learning method to verify the robustness of discriminating fecal samples from patients who did or did not develop BSI. Supervised learning using Random Forests accurately assigned samples to their source population based on taxonomic profiles at the family level (82.1 % accuracy or number of correct classifications divided by total number of classifications, 2.6 times better than the baseline error rate for random guessing). However, this was outperformed by the risk index approach according to leave-one-out cross-validation.

**Fig. 2:**
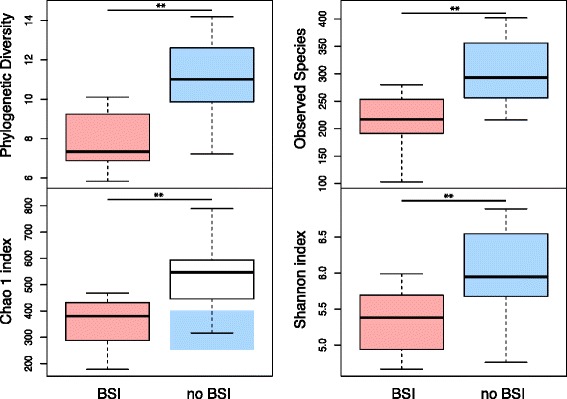
Alpha-diversity indices in samples collected prior to treatment in patients who developed subsequent BSI (*red*, *n* = 11) versus samples collected prior to treatment in patients who did not develop subsequent BSI (*blue*, *n* = 17), based on phylogenetic and non-phylogenetic richness. Analyses were performed on 16S rRNA V5 and V6 regions data, with a rarefaction depth of 3041 reads per sample. *Whiskers* in the *boxplot* represent the range of minimum and maximum alpha diversity values within a population, excluding outliers. Monte-Carlo permutation t-test: *p <0.05; **p <0.01; and ***p <0.001. *Boxplots* denote top quartile, median, and bottom quartile. BSI, Bloodstream infection. Patients who developed a subsequent BSI had significantly lower microbial richness compared with patients who did not develop subsequent BSI

Alpha diversity in fecal samples from patients who developed BSI was significantly lower than alpha diversity from patients who did not develop subsequent BSI, with reduced evenness (Shannon index, Monte Carlo permuted t-test two-sided *p* value = 0.004) and reduced richness (Observed species, Monte Carlo permuted t-test two-sided *p* value = 0.001) (Fig. 2). Further, these differences in richness between patients who developed BSI and patients who did not develop subsequent BSI are robust to rarefaction, being detected with as few as 500 reads per sample (Shannon index, Monte Carlo permuted t-test two-sided *p* value = 0.007; Observed species, Monte Carlo permuted t-test two-sided *p* value = 0.005, Additional file 4).

In order to determine whether differential sequencing depth between the BSI and non-BSI groups could be confounding our analysis by affecting diversity estimates resulting from OTU picking, we first verified that sequencing depth was not associated with BSI status (*p* = 0.9263, Mann–Whitney U test). Therefore, we do not expect sequencing depths to influence our results. We also subsampled the input sequences to achieve even depth per sample prior to performing OTU picking and then re-picked OTUs to determine whether differences in sequencing depth were affecting our OTU diversity. We did this at 1000, 2000, and 3000 sequences per sample. In each case, the groups remained significantly different (*p* <0.01, Mann–Whitney U test), with the BSI patients having lower diversity microbiomes in their pretreatment samples (Additional file 4).

### A novel microbiome-based BSI risk index predicts BSI

**Fig. 3:**
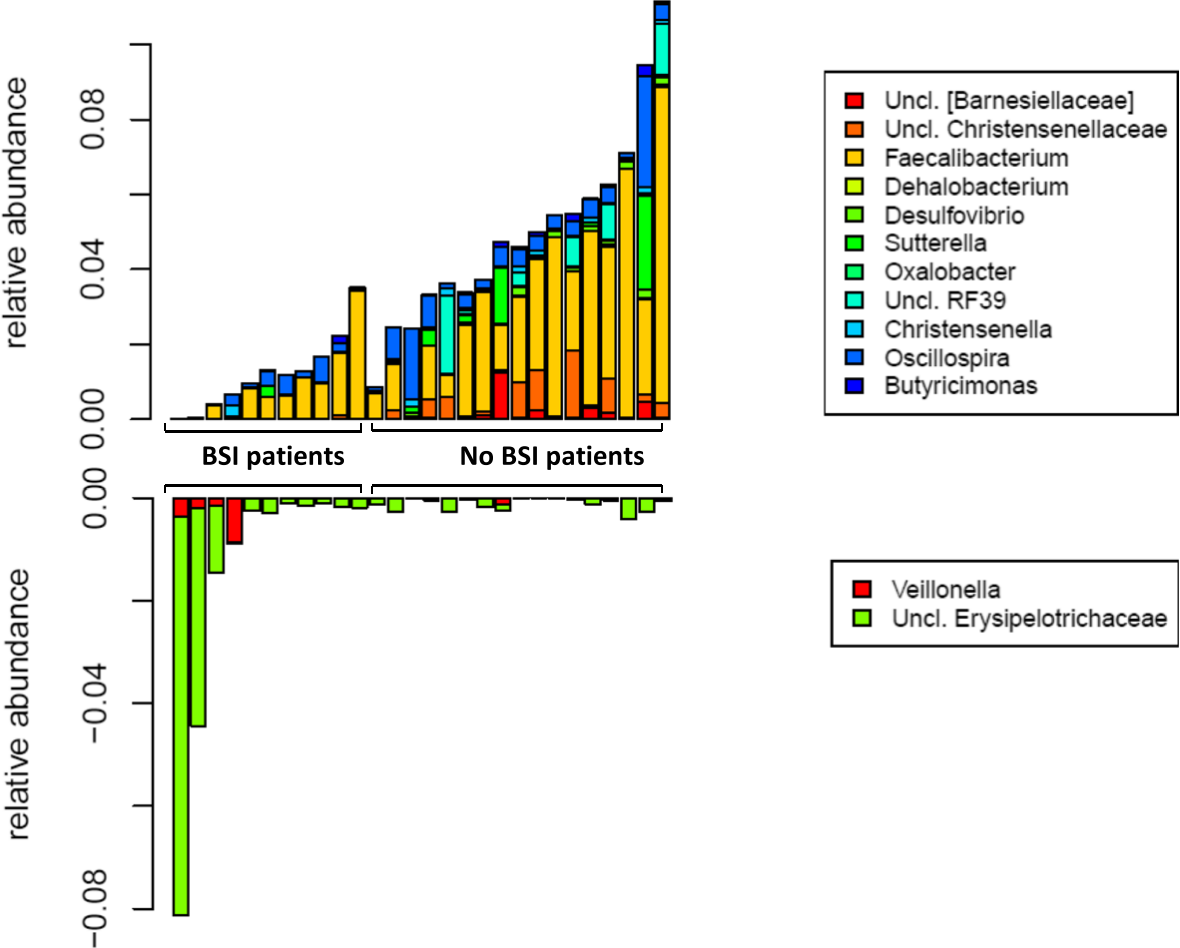
Relative abundance of the differentiated taxa in samples collected prior to treatment in patients who developed subsequent BSI (*n* = 11) and patients who did not develop BSI (*n* = 17). BSI, Bloodstream infection

We identified a panel of 13 microbes that were differentiated between patients who did and did not develop BSI (Mann–Whitney U test, FDR-corrected two-sided *p* value <0.15). Fecal samples collected prior to treatment from the patients who developed subsequent BSI exhibited significantly decreased abundance of members of Bacteroides (Barnesiellaceae, *Butyricimonas*), Firmicutes (Christensenellaceae, *Faecalibacterium*, *Oscillospira*, *Christensenella*, *Dehalobacterium*), Proteobacteria (*Desulfovibrio*, *Sutterella*, *Oxalobacter*) and Actinobacteria (Coriobacteriaceae) compared to patients who did not develop subsequent BSI. The patients who developed BSI exhibited significantly higher abundance of Erysipelotrichaceae and V*eillonella* in fecal samples collected prior to treatment compared to patients who did not develop subsequent BSI (Fig. 3, Additional files 5, 6, and 7).

We tested the individual ability of these microbes to discriminate between patients who did and did not develop subsequent BSI. Based on ROC curve analyses, we found that Barnesiellaceae yielded a ROC-plot AUC value of 0.94, Christensenellaceae yielded a ROC-plot AUC value of 0.86, and *Faecalibacterium* yielded a ROC-plot AUC value of 0.84 (Additional file 8).

**Fig. 4:**
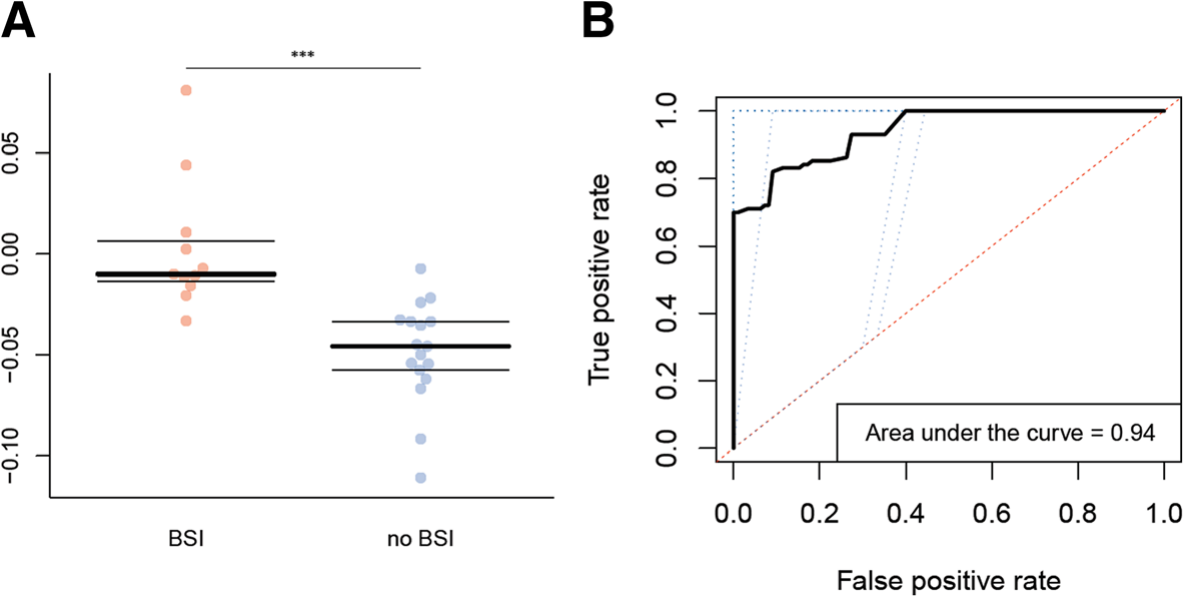
**a** BSI risk index based on the differentiated taxa (*n* = 28). We included in the BSI risk index all the taxa with a false discovery rate (FDR)-corrected *p* value less than 0.15. The BSI was then calculated using the sum of relative abundances of the taxa that were significantly associated with BSI minus the sum of the relative abundances of the taxa that were associated with protection from BSI. Mann–Whitney U test: ***p < 0.001. *Boxplots* denote top quartile, median, and bottom quartile. BSI, Bloodstream infection. **b** Receiving-operating characteristic (ROC) curve analysis of the BSI risk index in fecal samples collected prior to treatment, to differentiate patients who developed subsequent BSI and patients who did not develop BSI. We applied tenfold jack-knifing; the ten ROC curves are in *blue* and the mean ROC curve is in *black*. BSI, Bloodstream infection

To assess the predictive accuracy of this method for identifying the panel of bacteria, we then performed leave-one-out cross-validation, a rigorous statistical approach from machine learning, wherein the entire model is retrained on n-1 samples to predict the BSI risk of the held-out sample, and then the process is repeated for each sample. The predicted risk indices were highly differentiated between patients who did and did not develop BSI (Mann–Whitney U *p* value = 0.008). Median BSI risk index was −0.01 (IQR = 0.02) in patients who develop subsequent bacteremia and median BSI risk index was −0.05 (IQR = 0.02) in patients who did not develop BSI (Mann–Whitney U test, two-sided *p* value <0.001) (Fig. 4a). A negative risk index simply means that the protection-associated taxa were more abundant than the risk-associated bacteria, but not necessarily that the patient’s risk score was sufficiently low to be classified as low risk. ROC curve analysis showed that the BSI risk index was a strong predictor of the onset of subsequent BSI, with an AUC of 0.94 (Fig. 4b). In the leave-one-out classification, we determined that a BSI risk index classification threshold of −0.02 best predicts BSI in a new patient, yielding a sensitivity of 90 % at a specificity of 90 %. Importantly, the risk values shown in Fig. 4a are entirely predicted for each participant using a panel of microbes retrained from scratch only on the other participants. We then retrained the model one last time on the entire dataset to report the taxa included in the final predictive panel (Fig. 3).

### Clinical history does not predict BSI

Association between clinical data (age, sex, previous antibiotic treatment received, type of antibiotic treatment, delay of the previously received antibiotic treatment, previous chemotherapy received, and delay of the previously received chemotherapy) and BSI was tested using a univariate and a multivariate logistic regression with a backward step-wise procedure. No significant association was found between any clinical data and BSI (Additional file 9).

### Shifts in the microbiome functional repertoire in patients who developed subsequent BSI

We also predicted the functional composition of the fecal microbiome using PICRUSt. This algorithm estimates the functional potential of microbial communities given the current 16S rRNA gene survey and the set of currently sequenced reference genomes [15]. PICRUSt predictions in the human gut microbiome are expected to be 80–85 % correlated with the true metabolic pathway abundances. Therefore, the PICRUSt results should be considered suggestive only. We used LEfSe to identify significant differences in microbial genes (level 2 and level 3 KEGG Orthology groups, Linear Discriminant Analysis score (log10) >2) in the samples collected prior to treatment from patients who developed and did not develop subsequent BSI [18]. The fecal microbiome of patients that developed subsequent BSI were enriched in functional categories associated with xenobiotics biodegradation and metabolism and depleted in categories associated with transcription machinery, histidine metabolism, arginine and proline metabolism, lipid biosynthesis proteins and alanine, aspartate and glutamate metabolism (Additional file 10). Many of these alterations in the metabolic capacity were previously reported to compromise the intestinal epithelial barrier function, therefore potentially enabling bacterial translocation [19–22].

## Discussion

### Decreased diversity in pretreatment samples predicts BSI

A previous study found that mean measures of microbial diversity decreased over the course of HSCT [10]. Another recent study reported that reduced diversity, measured the day of the transplant, predicted the patients that will die during the HSCT procedure [23]. Reduced diversity of fecal microbiota in inflammatory states is well documented [24]. In a murine model of ileal Crohn’s disease (CD), induction of inflammation was associated with reduced microbial diversity and mucosal invasion by opportunistic pathogen [25]. Our findings provide further evidence that a diverse microbiome is associated with protection from BSI [26]. Furthermore, we demonstrate that decreases in gut microbial diversity are observed before patients even begin treatment. This suggests that certain patients may be predisposed to infection prior to entering the hospital and that we can identify these patients using their microbiota.

### Barnesiellaceae-enriched fecal microbiota is protective against BSI

In mice colonized with vancomycin-resistant Enterococcus (VRE), a recent study demonstrated that recolonization with microbiota that contain *Barnesiella* correlates with VRE clearance [27]. Moreover, in patients undergoing HSCT, intestinal colonization with *Barnesiella* was associated with resistance to Enterococcal domination, a risk factor for subsequent VRE BSI [10, 27]. Our findings support that this taxon is required to prevent expansion of oxygen-tolerant bacteria, such as *Enterococcus* and Enterobacteriaceae, the most frequent bloodstream pathogens in patients undergoing HSCT [28]. Barnesiellaceae was also decreased in patients with HIV compared to a healthy control group [29]. *Barnesiella* was found to be negatively correlated to TNF-α, markers of systemic inflammation in HIV patients [19]. Furthermore, *Barnesiella* was decreased in case of severe colitis in IL-22–deficient and co-housed wild-type mice, suggesting its protective role against inflammation [20]. In our findings, *Barnesiella* is an important member of the BSI protection-associated taxa, although there are several other taxa that are strongly associated with protection or risk of BSI.

### Ruminococceae-depleted fecal microbiota lead to BSI

*Faecalibacterium prauznitzii*, major member of the genus *Faecalibacterium*, is a well-described anti-inflammatory organism, considered to be a marker of GI health [24]. A recent study of cirrhotic patients showed that patients who presented a bacterial translocation had a lower ratio of *F. prausnitzii*/*E. coli* as compared to patients who did not have sepsis [21]. Additionally, *Oscillospira* was increased in microbiomes amended with *Christensenella minuta* for prevention of adiposity [30]. *Oscillospira* has also been reported to directly regulate components involved in the maintenance of gut barrier integrity [22]. Ruminococceae-modulated microbes were butyrate-producing bacteria. Butyrate is a short-chain fatty acid that has a key function in intestinal epithelium development [31]. Butyrate was previously reported to exhibit anti-inflammatory properties by reducing permeability of the intestinal epithelium. In addition, it has been proposed that butyrate can reinforce colonic defense barriers by increasing antimicrobial peptide levels and mucin production [9].

### Other BSI-protective taxa are associated with healthy states in published datasets

Christensenellaceae was enriched in fecal samples of healthy individuals when compared to pediatric and young adult IBD patients and in lean when compared to obese participants [30]. *Christensenella* was reported to be significantly depleted in fecal samples of ulcerative colitis patients [32], in fecal samples of patients with post-infectious irritable bowel syndrome [33], and in patients with CD relative to healthy controls [24]. A study demonstrated that *Desulfovibrio* is a common sulfate-reducing bacteria found in the fecal microbiota of healthy individuals, harboring positive effects on the gut barrier integrity [34]. The *Butyricimonas* genus, known as a butyrate producer with anti-inflammatory effects, was found decreased in the untreated multiple sclerosis patients as compared with healthy participants [35]. *Sutterella* was also found decreased in CD patients [24].

### BSI-associated taxa are linked to gut inflammation in published datasets

*Veillonella* has been previously associated with intestinal inflammation in CD patients [24]. Moreover, *Veillonella* was found enriched in in *Clostridium difficile* infection patients when compared to healthy controls [36]. Erysipelotrichaceae was described as one of the drivers of exacerbated intestinal inflammation in a mouse model of IBD [37]. Furthermore, in colorectal cancer patients and in a murine model of inflammation-associated colorectal cancer, Erysipelotrichaceae was associated with the inflammation and colonic tumorigenesis [38].

### Motivation for the predictive risk index model

The goal of a supervised learning method is to learn a function of some combination of predictors, such as the relative abundances of bacterial taxa, that correctly predicts an experimental outcome, such as BSI incidence. In microbiome data, this is a hard problem from a statistical perspective because the classifier must determine which taxa to include in the model and how much weight to assign to each taxon. Choosing which predictors to include from a large set of features is called feature selection. The problem becomes even more complicated when there are non-linear relationships between the taxa and the outcome, and when there are statistical dependencies between the taxa. Different types of classifiers have different levels of flexibility for incorporating these types of relationships. In general, the more parameters or degrees of freedom available to the classifier, the more flexible it is, but the larger the training set it requires to avoid over-fitting. Therefore, it is common to choose classifiers that have built-in constraints that keep them from being too flexible.

For example, if we were to fit a logistic regression to the relative abundances of all 176 genera observed in our data, using 27 of the 28 samples for training, the model would grossly overfit the training data and would not be likely to classify the held-out sample correctly on average. On the other hand, if we only based our model on the single most discriminative genus, then we would fail to account for inter-individual variation in genus membership and the potential for convergent evolution to allow different taxa to perform the same functions in different people, and again we would not expect good predictive performance. The goal is to find a good method that is neither too flexible (too many degrees of freedom) nor too constrained (too few degrees of freedom). A common solution to the problem of over-fitting is to force most of the regression coefficients to be very small by constraining their sum-of-squares or their sum-of-absolute-values to be less than a particular threshold. However, determining the correct threshold requires the use of a nested cross-validation procedure. In this and other recent analyses, we have found that a simple approach to feature selection using the univariate Mann–Whitney U test does a good job of identifying useful predictors without the need for nested cross-validation to tune model parameters.

Furthermore, once a subset of predictors has been identified, in smaller datasets it may be challenging statistically to learn the correct regression coefficients for each of the predictors. Instead, we reasoned that in the absence of sufficient data for determining proper regression coefficients, a good proxy for the strength of an association between a taxon and a clinical phenotype of the host is simply its relative abundance. Therefore, we chose to use the additive risk index as our predictive model, which is equivalent to a linear model in which all of the regression coefficients are 1 (for risk-associated taxa), −1 (for protection-associated taxa), or 0 (for taxa not identified as significant using the Mann–Whitney U test). This approach is consistent with the theory of convergent evolution, in which multiple different species may be occupying the same ecological niche in different human individuals, under the assumption that the niche population sizes are relatively consistent across species. Another benefit is that, in contrast to a ratio-based risk index, the additive index can easily produce meaningful scores when a patient is completely lacking either the protection-associated taxa or the risk-associated taxa. It is important to note that the larger the microbiome dataset, the more likely it is that a more complex classifier will provide better predictive accuracy on held-out data. However, many clinical microbiome datasets are still limited in size due to limitations of patient recruitment and funding, in which case the additive risk index may be a useful alternative to more complex and more flexible supervised learning models.

### Alternatives to fecal-microbiota transplant therapy in immunocompromised patients

Our findings demonstrate that there is a predictive relationship between pre-chemotherapy gut microbiome and future risk of BSI in patients with NHL receiving allogeneic transplantation. To the extent that gut microbiome does contribute to BSI risk, future management of patients submitted to HSCT procedure may include administration of microbiome-targeting therapeutics to decrease risk of infectious complications. One obvious strategy would be fecal microbiota transplantation from a healthy donor or even from preserved donation of the patient’s own microbiota. However, this therapeutic approach may lead to exposure to unknown pathogens and/or potential transfer of a risk-associated microbiota, not to mention microbiota that may predispose the recipient to various microbiome-linked diseases [39]. Therefore, we proposed an alternative strategy: to select a consortium of OTUs expected have protective and beneficial effects on the host that could be administered to the patients during the HSCT procedure. A clear next step is to evaluate a consortium of microbial taxa for its ability to prevent or decrease risk of BSI.

Our study has several limitations. First, our cohort is limited to patients with NHL receiving allogeneic HSCT. Thus, our BSI risk index prediction may not be generalizable to other chemotherapy regimens, other hematological malignancies, and other immunocompromised patients, although it is suggestive that similar approaches could apply in those populations. The next step will be to validate the performance of the BSI risk index presented here in a larger cohort of patients with other hematological malignancies and receiving different types of chemotherapy regimen. Second, patients received various cancer-specific treatments before HSCT procedure that may affect pre-HSCT microbiome composition, although we did not find an association between clinical history and BSI risk. Third, sequence coverage per sample was somewhat low for one sample (3041 sequences), although a previous study showed that large effects can be recovered with as few as 100 or even 10 sequences per sample [40]. Here we showed that alpha- and beta-diversity findings were retained even when subsampling data were down to very shallow depths of 500 sequences per sample. To avoid throwing out data contained in the higher depth samples for the taxon association and risk index analyses, we used the normalized relative abundances from full-depth samples in place of rarefied data.

## Conclusions

Identifying cancer patients at high risk for BSI is a significant clinical challenge and is an important step toward reducing morbidity and mortality during the early transplant period. Our 16S rRNA gene sequencing-based analysis showed that a significant shift in microbial community structure precedes BSI, even before chemotherapy begins. Our findings also suggest the possibility of preventive manipulation of the intestinal microbiota to reduce risk of life-threatening infection in immunocompromised patients undergoing HCST. Based on our results we recommend future research into the development of a microbiome-targeted therapy to prevent BSI.

### Study approval

Written informed consent was obtained from all patients. The protocol received IRB approval by the Nantes University Hospital Ethics Committee. This study conformed to the Helsinki Declaration and to local legislation.

### Availability of data and materials

The datasets (16S rRNA sequences) supporting the conclusions of this article have been deposited at the National Center for Biotechnology Information as BioProject with top-level umbrella project ID PRJNA257960 and SRA experiment ID SRX733464.

## Additional files

Additional file 1: Code used in R to build the BSI risk index. (PDF 175 kb) Additional file 2: Sequence coverage per sample. (PDF 200 kb) Additional file 3: Association using a linear model of antibiotic prophylaxis and microbiome composition collapsed at genus level. (PDF 13 kb) Additional file 4: Alpha-diversity indices in samples collected prior to treatment in patients who developed subsequent BSI (*red*, *n* = 11) versus samples collected prior to treatment in patients who did not develop subsequent BSI (*blue*, *n* = 17), based on phylogenetic and non-phylogenetic richness. Analyses were performed on 16S rRNA V5 and V6 regions data, with a rarefaction depth of 500 reads per sample. *Whiskers* in the *boxplot* represent the range of minimum and maximum alpha diversity values within a population, excluding outliers. Monte-Carlo permutation t-test: **p* <0.05; ***p* <0.01; and ****p* <0.001. *Boxplots* denote top quartile, median, and bottom quartile. BSI, Bloodstream infection. Patients who developed a subsequent BSI had significantly lower microbial richness compared with patients who did not develop subsequent BSI. Similar *boxplots* are shown in Additional file 4, Fig. 2 using re-picked OTUs after subsampling sequence data to 1000, 2000, and 3000 sequences per sample. (PDF 110 kb) Additional file 5:
**A** Families that differ between fecal samples collected prior to treatment in patients who did or did not develop BSI (Mann–Whitney U test, False Discovery Rate-corrected, or FDR-corrected). BSI, Bloodstream infection. **B** Genera that differ between fecal samples collected prior to treatment in patients who did or did not develop BSI (Mann–Whitney U test, FDR-corrected *p* value). BSI, Bloodstream infection. (PDF 25 kb) Additional file 6: Taxonomic profile of the gut microbiomes of the samples collected prior to treatment in patients who developed subsequent BSI (*n* = 11) and in patients who did not develop subsequent BSI (*n* = 17). Analyses were performed on 16S rRNA V5 and V6 regions data, with a rarefaction depth of 3041 reads per sample. Relative taxa abundance plots for individuals from the samples collected before chemotherapy in patients who developed subsequent BSI and in patients who did not develop subsequent BSI, summarized at the family level. Individuals are represented along the horizontal axis and relative taxa frequency is denoted by the vertical axis. BSI, Bloodstream infection. (PDF 94 kb) Additional file 7: Relative abundance of the most significant taxa in samples collected prior to treatment in patients who developed subsequent BSI (*n* = 11) and patients who did not develop BSI (*n* = 17). Mann–Whitney test: **p* <0.05; ***p* <0.01; ****p* <0.001. *Boxplots* denote top quartile, median, and bottom quartile. BSI, Bloodstream infection. (PDF 23 kb) Additional file 8: Receiving-operating characteristic (ROC) curves analyses of the most distinctive taxa, in fecal samples collected prior to treatment, to differentiate patients who developed subsequent BSI and patients who did not develop BSI. We applied a tenfold jack-knifing, the ten ROC curves are in *blue* and the mean ROC curve is in *black*. BSI, Bloodstream infection. (PDF 100 kb) Additional file 9: Association between clinical characteristics of the patients and the outcome (BSI) based on a logistic regression model with backward elimination. BSI, Bloodstream infection. (PDF 95 kb) Additional file 10:
**A** Most significantly altered metabolic pathways (L2 and L3 KEGG) in samples collected prior to treatment between patients who developed subsequent BSI (*n* = 11) and patients who did not develop BSI (*n* = 17). Mann–Whitney test: **p* <0.05. *Boxplots* denote top quartile, median, and bottom quartile. **B** Linear Discriminant Analysis scores of differentially abundant microbial genes in gut microbiomes associated with or without subsequent BSI in fecal samples collected prior chemotherapy. (PDF 95 kb)

## Abbreviations

BSI: Bloodstream infection
HSTC: Hematopoietic stem cell transplantation
ICU: Intensive Care Unit
NHL: Non-Hodgkin lymphoma
OTU: Operational taxonomic unit
