# Erratum to: Pretreatment gut microbiome predicts chemotherapy-related bloodstream infection

**DOI:** 10.1186/s13073-016-0321-0

**Published:** 2016-05-26

**Authors:** Emmanuel Montassier, Gabriel A. Al-Ghalith, Tonya Ward, Stephane Corvec, Thomas Gastinne, Gilles Potel, Philippe Moreau, Marie France De La Cochetiere, Eric Batard, Dan Knights

**Affiliations:** Université de Nantes, EA 3826 Thérapeutiques cliniques et expérimentales des infections, Faculté de médecine, 1 Rue G Veil, Nantes, 44000 France; Department of Computer Science and Engineering, University of Minnesota, Minneapolis, MN 55455 USA; Biomedical Informatics and Computational Biology, University of Minnesota, Minneapolis, MN 55455 USA; Biotechnology Institute, University of Minnesota, St. Paul, MN 55108 USA; Nantes University Hospital, Microbiology Laboratory, Nantes, France; Hematology Department, Nantes University Hospital, Nantes, France

## Erratum

It has come to our attention that there is an error in one of the author names for this article [1]. The author name Philippe Moreau was incorrectly spelt as Phillipe Moreau. The original article has now been updated and the publisher apologises for any inconvenience caused.
