# Supplementary material for: Pretreatment gut microbiome predicts chemotherapy-related bloodstream infection
Source: Genome Med. 2016 Apr 28;8:49. doi: 10.1186/s13073-016-0301-4 (PMC4848771; doi:10.1186/s13073-016-0301-4)
Supplement: Additional file 1: — Code used in R to build the BSI risk index. (PDF 175 kb) [file 13073_2016_301_MOESM1_ESM.pdf]

Additional file 1. Code used in R to build the BSI risk index.

```
#read the mapping file and the otu table generated with QIIME

our.x <- t(read.table('wf_taxa_summary/otu_table_L6.txt',sep='\t',head=T,row=1,check=F))

our.map <- read.table('mapping-file.txt',sep='\t',head=T,row=1,check=F,comment='')

#perform the MWU test

our.x <- our.x[rownames(our.map),]

pvals <- apply(our.x,2,function(xx) wilcox.test(xx ~ our.map$Treatment,exact=F)$p.value)

pvals <- p.adjust(pvals,'fdr')

# determine good and bad bugs

hit.ix <- pvals < alpha

bsi.means <- colMeans(our.x[our.map$Treatment == 'bacteremia',])

hc.means <- colMeans(our.x[our.map$Treatment == 'nobacteremia',])

good.bugs <- hit.ix & ((hc.means - bsi.means) > 0)

bad.bugs <- hit.ix & ((hc.means - bsi.means) < 0)

#obtain the risk index

risk.index <- rowSums(our.x[,bad.bugs]) - rowSums(our.x[,good.bugs])
```
