# Supplementary material for: Pretreatment gut microbiome predicts chemotherapy-related bloodstream infection
Source: Genome Med. 2016 Apr 28;8:49. doi: 10.1186/s13073-016-0301-4 (PMC4848771; doi:10.1186/s13073-016-0301-4)
Supplement: Additional file 2: — Sequence coverage per sample. (PDF 200 kb) [file 13073_2016_301_MOESM2_ESM.pdf]

Additional file 2. Sequence coverage per sample.

Number of samples: 28

Number of observations: 3857

Total count: 280416

Counts per sample summary:

Min: 3041.0

Max: 26122.0

Median: 8830.500

Mean: 10014.857

Std. dev.: 4295.663

Counts per sample detail:

a11: 3041.0 (BSI)

a27: 5403.0 (no BSI)

a47: 6351.0 (no BSI)

a07: 6578.0 (BSI)

a28: 7194.0 (no BSI)

a46: 7260.0 (no BSI)

a23: 7570.0 (no BSI)

a38: 8047.0 (BSI)

a21: 8317.0 (BSI)

a29: 8334.0 (no BSI)

a30: 8378.0 (no BSI)

a50: 8581.0 (BSI)

a34: 8735.0 (no BSI)

a24: 8824.0 (BSI)

a33: 8837.0 (no BSI)

a35: 9299.0 (BSI)

a51: 9574.0 (no BSI)

a26: 10417.0 (BSI)

a39: 10646.0 (no BSI)

a40: 10897.0 (no BSI)

a43: 11183.0 (no BSI)

a44: 11392.0 (no BSI)

a55: 11756.0 (no BSI)

a52: 11827.0 (BSI)

a37: 13124.0 (BSI)

a14: 13211.0 (BSI)

a57: 19518.0 (no BSI)

a41: 26122.0 (no BSI)
