# Supplementary material for: Pretreatment gut microbiome predicts chemotherapy-related bloodstream infection
Source: Genome Med. 2016 Apr 28;8:49. doi: 10.1186/s13073-016-0301-4 (PMC4848771; doi:10.1186/s13073-016-0301-4)
Supplement: Additional file 3: — Association using a linear model of antibiotic prophylaxis and microbiome composition collapsed at genus level. (PDF 13 kb) [file 13073_2016_301_MOESM3_ESM.pdf]

Covariate	Taxon	P value	FDR-corrected P value	coefficient
Previous use of penicillin V	k__Bacteria;p__Firmicutes;c__Clostridia;o__Clostridiales;Other;Other	0.01566264	0.764250581	0.010290104
Previous use of penicillin V	k__Bacteria;p__Bacteroidetes;c__Flavobacteriia;o__Flavobacteriales;f__Flavobacteriaceae;g__	0.028120169	0.764250581	-4.86E-005
Previous use of penicillin V	k__Bacteria;p__Firmicutes;c__Clostridia;o__Clostridiales;f__Ruminococcaceae;g__Oscillospira	0.029987886	0.764250581	0.001915854
Previous use of penicillin V	k__Bacteria;p__Proteobacteria;c__Gammaproteobacteria;o__Enterobacteriales;f__Enterobacteriaceae;g__Citrobacter	0.030213391	0.764250581	-0.000140014
Previous use of penicillin V	k__Bacteria;p__Firmicutes;c__Erysipelotrichi;o__Erysipelotrichales;f__Erysipelotrichaceae;g__	0.033937694	0.764250581	-0.001439864
**Covariate**	**Taxon**	**P value**	**FDR-corrected P value**	**coefficient**
Previous use of cotrimoxazole	k__Bacteria;p__Firmicutes;c__Clostridia;o__Clostridiales;Other;Other	0.016597538	0.738446396	-0.009248303
Previous use of cotrimoxazole	k__Bacteria;p__Firmicutes;c__Erysipelotrichi;o__Erysipelotrichales;f__Erysipelotrichaceae;g__Coprobacillus	0.019250528	0.738446396	3.78E-005
Previous use of cotrimoxazole	k__Bacteria;p__Firmicutes;c__Bacilli;o__Lactobacillales;f__Leuconostocaceae;g__Leuconostoc	0.02003151	0.738446396	0.000262926
Previous use of cotrimoxazole	k__Bacteria;p__Proteobacteria;c__Betaproteobacteria;o__Burkholderiales;f__Oxalobacteraceae;g__	0.022648094	0.738446396	-5.88E-005
Previous use of cotrimoxazole	k__Bacteria;p__Bacteroidetes;c__Bacteroidia;o__Bacteroidales;f__Rikenellaceae;Other	0.03776668	0.738446396	-2.42E-005

Additional file 3. Association using a linear model of antibiotic prophylaxis and microbiome composition collapsed at genus level
