# Supplementary material for: Pretreatment gut microbiome predicts chemotherapy-related bloodstream infection
Source: Genome Med. 2016 Apr 28;8:49. doi: 10.1186/s13073-016-0301-4 (PMC4848771; doi:10.1186/s13073-016-0301-4)
Supplement: Additional file 4: — Alpha-diversity indices in samples collected prior to treatment in patients who developed subsequent BSI (red, n = 11) versus samples collected prior to treatment in patients who did not develop subsequent BSI (blue, n = 17), based on phylogenetic and non-phylogenetic richness. Analyses were performed on 16S rRNA V5 and V6 regions data, with a rarefaction depth of 500 reads per sample. Whiskers in the boxplot represent the range of minimum and maximum alpha diversity values within a population, excluding outliers. Monte-Carlo permutation t-test: *p <0.05; **p <0.01; and ***p <0.001. Boxplots denote top quartile, median, and bottom quartile. BSI, Bloodstream infection. Patients who developed a subsequent BSI had significantly lower microbial richness compared with patients who did not develop subsequent BSI. Similar boxplots are shown in Additional file 4, Fig. 2 using re-picked OTUs after subsampling sequence data to 1000, 2000, and 3000 sequences per sample. (PDF 110 kb) [file 13073_2016_301_MOESM4_ESM.pdf]

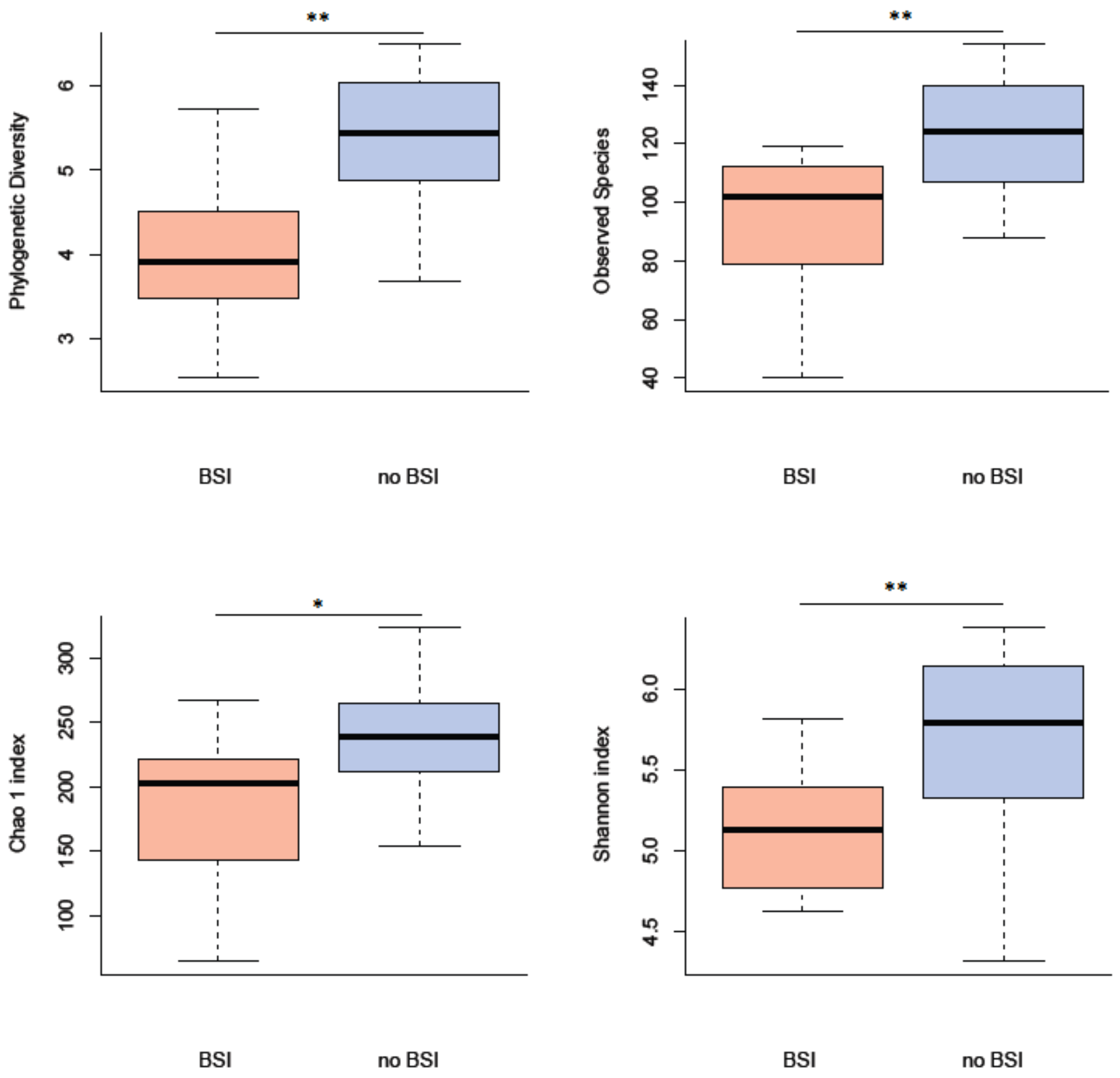

Additional File 4, Figure 1. Observed differences in diversity persist after rarefaction of OTU counts at 500 observations per sample.

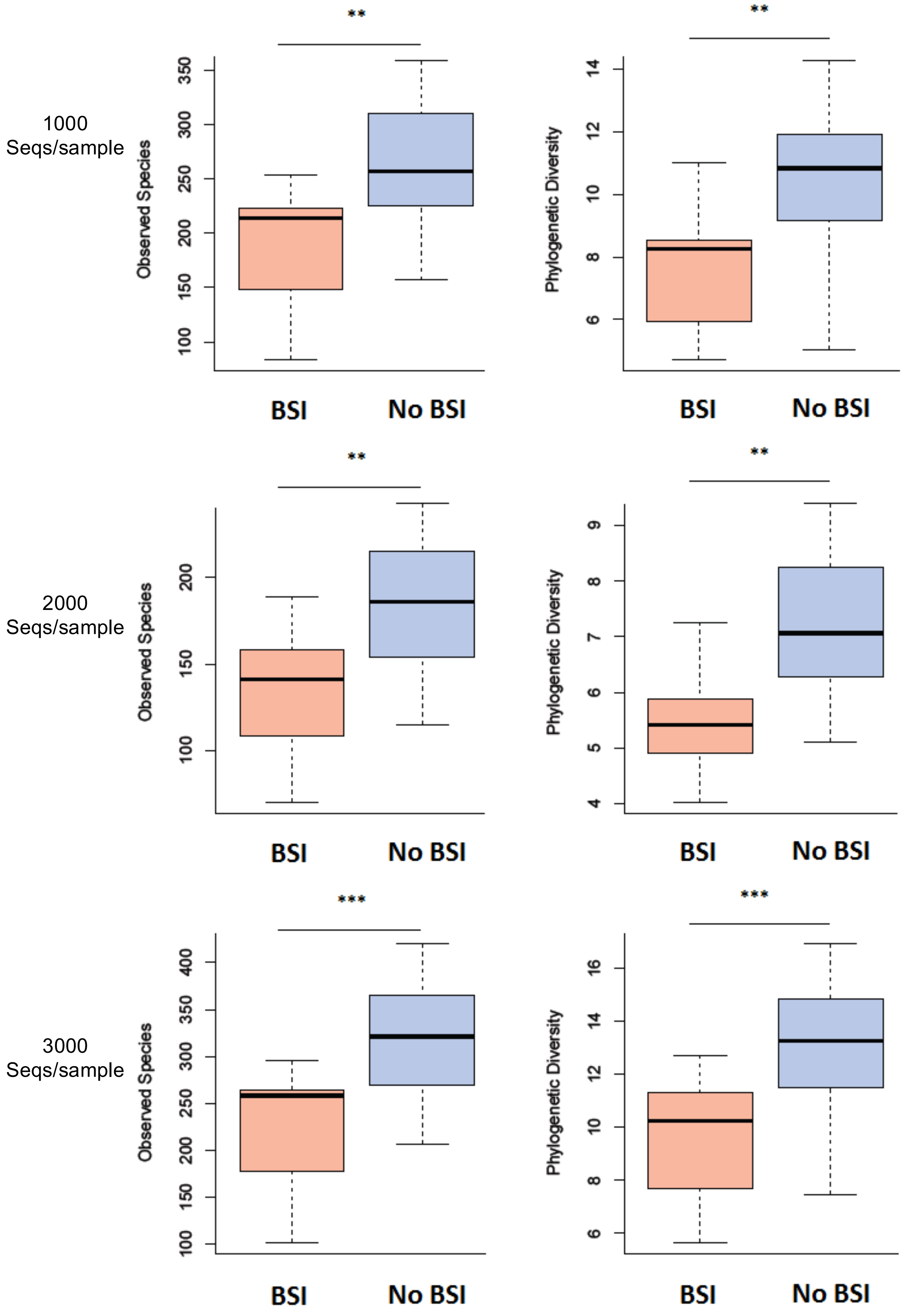

Additional File 4, Figure 2. Observed differences in diversity persist after re-picking OTUs using even depths of 1,000, 2,000, and 3,000 sequences per sample.
