# Supplementary material for: Pretreatment gut microbiome predicts chemotherapy-related bloodstream infection
Source: Genome Med. 2016 Apr 28;8:49. doi: 10.1186/s13073-016-0301-4 (PMC4848771; doi:10.1186/s13073-016-0301-4)
Supplement: Additional file 5: — A Families that differ between fecal samples collected prior to treatment in patients who did or did not develop BSI (Mann–Whitney U test, False Discovery Rate-corrected, or FDR-corrected). BSI, Bloodstream infection. B Genera that differ between fecal samples collected prior to treatment in patients who did or did not develop BSI (Mann–Whitney U test, FDR-corrected p value). BSI, Bloodstream infection. (PDF 25 kb) [file 13073_2016_301_MOESM5_ESM.pdf]

**Feature**	**P value**	**FDR-corrected P value**	**Mean in BSI samples**	**Mean in no BSI samples**
k__Bacteria;p__Bacteroidetes;c__Bacteroidia;o__Bacteroidales;f__[Barnesiellaceae]	4.80123E-05	0.002160555	0	0.030608691
k__Bacteria;p__Firmicutes;c__Clostridia;o__Clostridiales;f__Christensenellaceae	0.000976047	0.021961056	0.017263335	0.061397059
k__Bacteria;p__Proteobacteria;c__Deltaproteobacteria;o__Desulfovibrionales;f__Desulfovibrionaceae	0.002583052	0.038745776	0.009117493	0.035661785
k__Bacteria;p__Firmicutes;c__Clostridia;o__Clostridiales;f__Dehalobacteriaceae	0.005454802	0.061366525	0	0.008590271
k__Bacteria;p__Proteobacteria;c__Betaproteobacteria;o__Burkholderiales;f__Alcaligenaceae	0.008700052	0.077199038	0.00824569	0.0361082
k__Bacteria;p__Tenericutes;c__Mollicutes;o__RF39;f__	0.010293205	0.077199038	0	0.033369899
k__Bacteria;p__Proteobacteria;c__Betaproteobacteria;o__Burkholderiales;f__Oxalobacteraceae	0.015134529	0.085608235	0.003111599	0.011823771
k__Bacteria;p__Bacteroidetes;c__Bacteroidia;o__Bacteroidales;f__[Odoribacteraceae]	0.015219242	0.085608235	0.01364343	0.037690691
k__Bacteria;p__Firmicutes;c__Bacilli;o__Lactobacillales;f__Lactobacillaceae	0.036943059	0.157390604	0.111370127	0.017814454
k__Bacteria;p__Firmicutes;c__Clostridia;o__Clostridiales;f__	0.038473259	0.157390604	0.142158954	0.238485423
k__Bacteria;p__Firmicutes;c__Clostridia;o__Clostridiales;f__Ruminococcaceae	0.038473259	0.157390604	0.36528884	0.462636458

Additional file 5 A) Families that differ between fecal samples collected prior to treatment in patients who did or did not develop BSI. (Mann-Whitney U test, False Discovery Rate (FDR)-corrected). BSI : Bloodstream Infection.

Feature	P value	FDR-corrected P value	Mean in BSI samples	Mean in no BSI samples
k__Bacteria;p__Bacteroidetes;c__Bacteroidia;o__Bacteroidales;f__[Barnesiellaceae];g__	4.80123E-05	0.0035049	0	0.030608691
k__Bacteria;p__Firmicutes;c__Clostridia;o__Clostridiales;f__Christensenellaceae;g__	0.002715736	0.093925496	0.009486937	0.051637282
k__Bacteria;p__Firmicutes;c__Clostridia;o__Clostridiales;f__Ruminococcaceae;g__Faecalibacterium	0.004106589	0.093925496	0.075943204	0.157567367
k__Bacteria;p__Firmicutes;c__Clostridia;o__Clostridiales;f__Dehalobacteriaceae;g__Dehalobacterium	0.005454802	0.093925496	0	0.008110837
k__Bacteria;p__Proteobacteria;c__Deltaproteobacteria;o__Desulfovibrionales;f__Desulfovibrionaceae;g__Desulfovibrio	0.006485006	0.093925496	0.001185782	0.022130464
k__Bacteria;p__Proteobacteria;c__Betaproteobacteria;o__Burkholderiales;f__Alcaligenaceae;g__Sutterella	0.008700052	0.093925496	0.00824569	0.0361082
k__Bacteria;p__Proteobacteria;c__Betaproteobacteria;o__Burkholderiales;f__Oxalobacteraceae;g__Oxalobacter	0.010293205	0.093925496	0	0.008363649
k__Bacteria;p__Tenericutes;c__Mollicutes;o__RF39;f__;g__	0.010293205	0.093925496	0	0.033369899
k__Bacteria;p__Firmicutes;c__Clostridia;o__Clostridiales;f__Veillonellaceae;g__Veillonella	0.014371074	0.116565377	0.023380631	0.002898584
k__Bacteria;p__Firmicutes;c__Clostridia;o__Clostridiales;f__Christensenellaceae;g__Christensenella	0.016143634	0.117848525	0.012657346	0.026329171
k__Bacteria;p__Firmicutes;c__Clostridia;o__Clostridiales;f__Ruminococcaceae;g__Oscillospira	0.018671821	0.118013149	0.039894856	0.072200504
k__Bacteria;p__Bacteroidetes;c__Bacteroidia;o__Bacteroidales;f__[Odoribacteraceae];g__Butyricimonas	0.019399422	0.118013149	0.005621577	0.018437991
k__Bacteria;p__Firmicutes;c__Erysipelotrichi;o__Erysipelotrichales;f__Erysipelotrichaceae;g__	0.02394951	0.134485712	0.082986672	0.029542167

Additional file 5 B) Genera that differ between fecal samples collected prior to treatment in patients who did or did not develop BSI. (Mann-Whitney U test, False Discovery Rate (FDR)-corrected). BSI : Bloodstream Infection.
