# Supplementary figures and images for: Pretreatment gut microbiome predicts chemotherapy-related bloodstream infection
Source: Genome Med. 2016 Apr 28;8:49. doi: 10.1186/s13073-016-0301-4 (PMC4848771; doi:10.1186/s13073-016-0301-4)

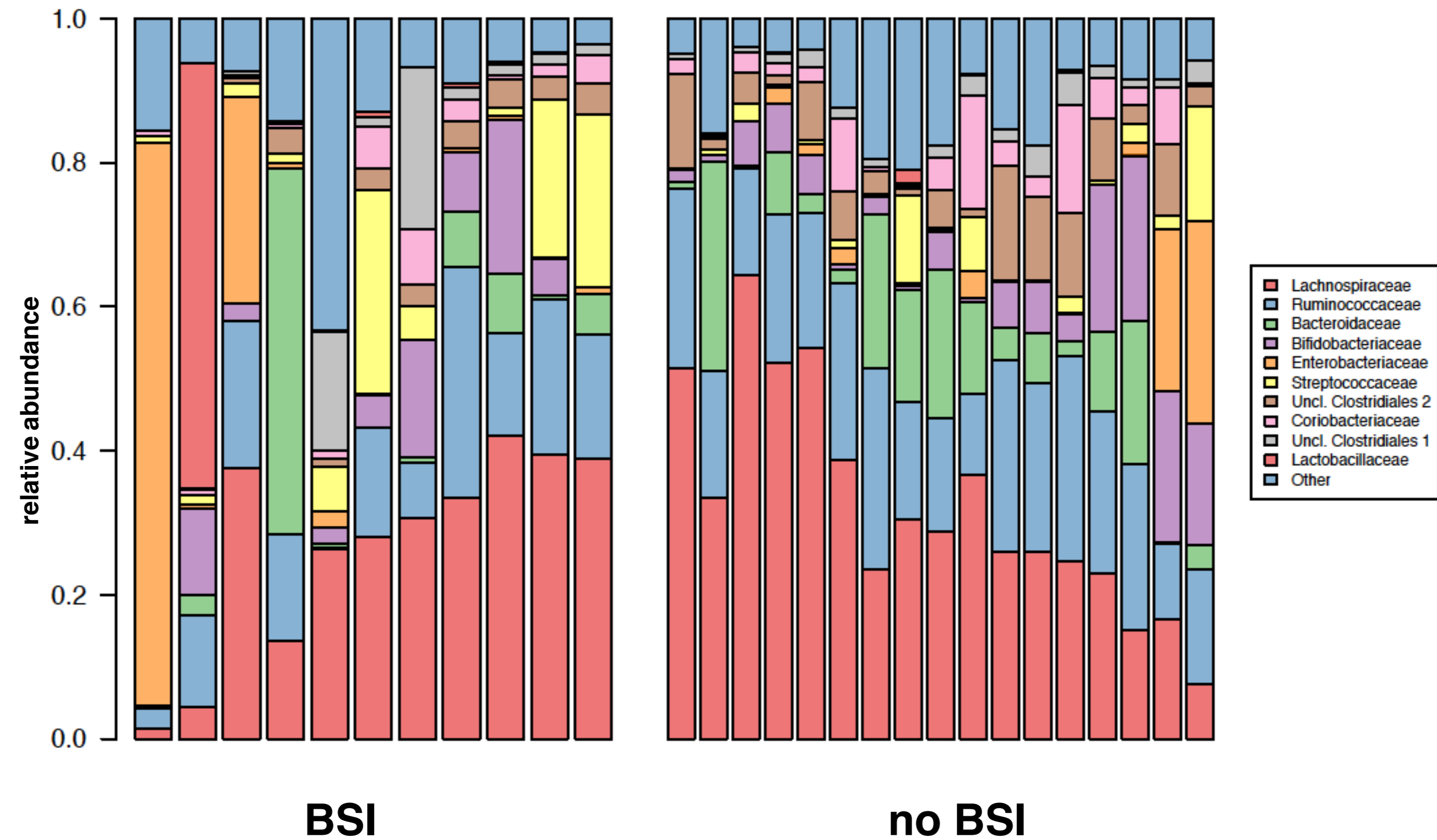

Supplement: Additional file 6: — Taxonomic profile of the gut microbiomes of the samples collected prior to treatment in patients who developed subsequent BSI (n = 11) and in patients who did not develop subsequent BSI (n = 17). Analyses were performed on 16S rRNA V5 and V6 regions data, with a rarefaction depth of 3041 reads per sample. Relative taxa abundance plots for individuals from the samples collected before chemotherapy in patients who developed subsequent BSI and in patients who did not develop subsequent BSI, summarized at the family level. Individuals are represented along the horizontal axis and relative taxa frequency is denoted by the vertical axis. BSI, Bloodstream infection. (PDF 94 kb) [file 13073_2016_301_MOESM6_ESM.pdf]
