# Supplementary material for: Pretreatment gut microbiome predicts chemotherapy-related bloodstream infection
Source: Genome Med. 2016 Apr 28;8:49. doi: 10.1186/s13073-016-0301-4 (PMC4848771; doi:10.1186/s13073-016-0301-4)
Supplement: Additional file 7: — Relative abundance of the most significant taxa in samples collected prior to treatment in patients who developed subsequent BSI (n = 11) and patients who did not develop BSI (n = 17). Mann–Whitney test: *p <0.05; **p <0.01; ***p <0.001. Boxplots denote top quartile, median, and bottom quartile. BSI, Bloodstream infection. (PDF 23 kb) [file 13073_2016_301_MOESM7_ESM.pdf]

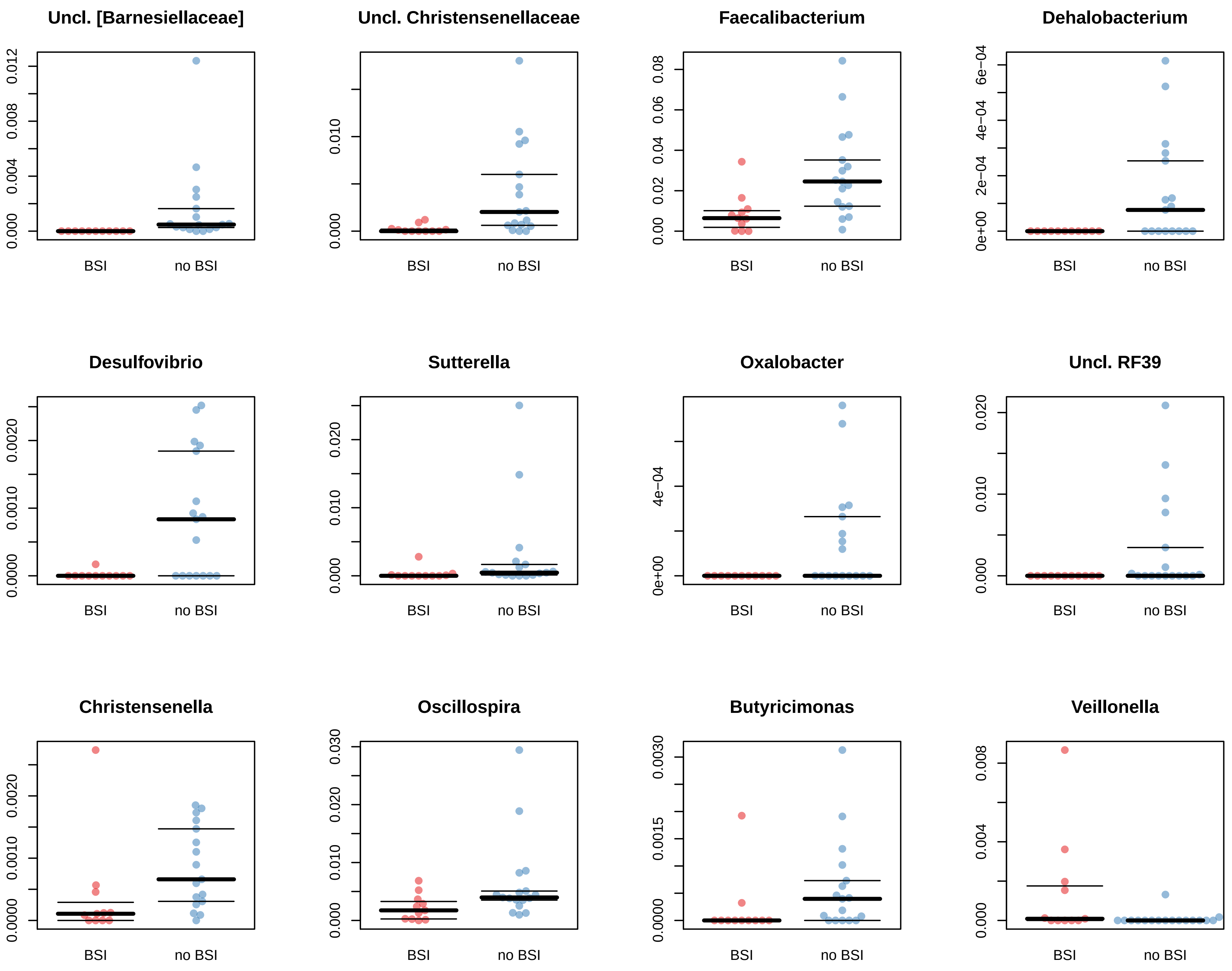
Uncl. [Barnesiellaceae]
Uncl. Christensenellaceae
Faecalibacterium
Dehalobacterium
Desulfovibrio
Sutterella
Oxalobacter
Uncl. RF39
Christensenella
Oscillospira
Butyricimonas
Veillonella
BSI
no BSI
