# Supplementary material for: Pretreatment gut microbiome predicts chemotherapy-related bloodstream infection
Source: Genome Med. 2016 Apr 28;8:49. doi: 10.1186/s13073-016-0301-4 (PMC4848771; doi:10.1186/s13073-016-0301-4)
Supplement: Additional file 8: — Receiving-operating characteristic (ROC) curves analyses of the most distinctive taxa, in fecal samples collected prior to treatment, to differentiate patients who developed subsequent BSI and patients who did not develop BSI. We applied a tenfold jack-knifing, the ten ROC curves are in blue and the mean ROC curve is in black. BSI, Bloodstream infection. (PDF 100 kb) [file 13073_2016_301_MOESM8_ESM.pdf]

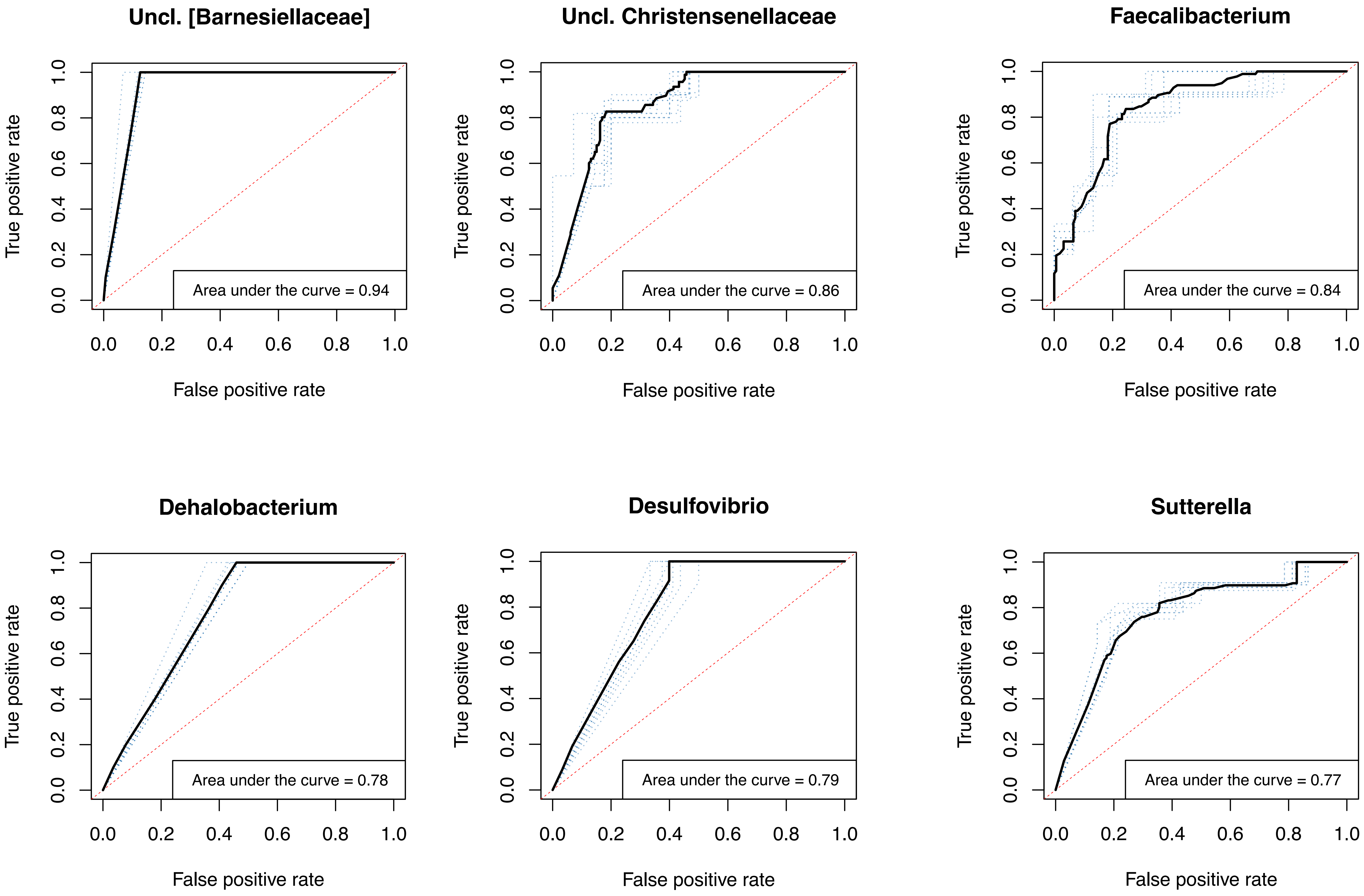
Uncl. [Barnesiellaceae]
Area under the curve = 0.94
Uncl. Christensenellaceae
Area under the curve = 0.86
Faecalibacterium
Area under the curve = 0.84
Dehalobacterium
Area under the curve = 0.78
Desulfovibrio
Area under the curve = 0.79
Sutterella
Area under the curve = 0.77
True positive rate
False positive rate
0.0
0.2
0.4
0.6
0.8
1.0
