# Supplementary material for: Pretreatment gut microbiome predicts chemotherapy-related bloodstream infection
Source: Genome Med. 2016 Apr 28;8:49. doi: 10.1186/s13073-016-0301-4 (PMC4848771; doi:10.1186/s13073-016-0301-4)
Supplement: Additional file 9: — Association between clinical characteristics of the patients and the outcome (BSI) based on a logistic regression model with backward elimination. BSI, Bloodstream infection. (PDF 95 kb) [file 13073_2016_301_MOESM9_ESM.pdf]

	**Estimate**	**Standart Error**	**P-value**
**(Intercept)**	23.50988	2399.54961	0.9922
**Antibiotic prophylaxis penicillin V**	-1.48463	1.09895	0.1767
**Antibiotic prophylaxis Cotrimoxazole**	1.63217	1.03467	0.1147
**Chemotherapy previous use**	-18.76785	2399.54562	0.9938
**Delay of the Chemotherapy**	-3.88820	2.14295	0.0696
**Age of the patient**	-0.07561	0.05462	0.1663

Additional file 9. Association between clinical characteristics of the patients and the outcome (BSI) based on a logistic regression model with backward elimination. BSI: Bloodstream Infection.
