# Supplementary material for: Pretreatment gut microbiome predicts chemotherapy-related bloodstream infection
Source: Genome Med. 2016 Apr 28;8:49. doi: 10.1186/s13073-016-0301-4 (PMC4848771; doi:10.1186/s13073-016-0301-4)
Supplement: Additional file 10: — A Most significantly altered metabolic pathways (L2 and L3 KEGG) in samples collected prior to treatment between patients who developed subsequent BSI (n = 11) and patients who did not develop BSI (n = 17). Mann–Whitney test: *p <0.05. Boxplots denote top quartile, median, and bottom quartile. B Linear Discriminant Analysis scores of differentially abundant microbial genes in gut microbiomes associated with or without subsequent BSI in fecal samples collected prior chemotherapy. (PDF 95 kb) [file 13073_2016_301_MOESM10_ESM.pdf]

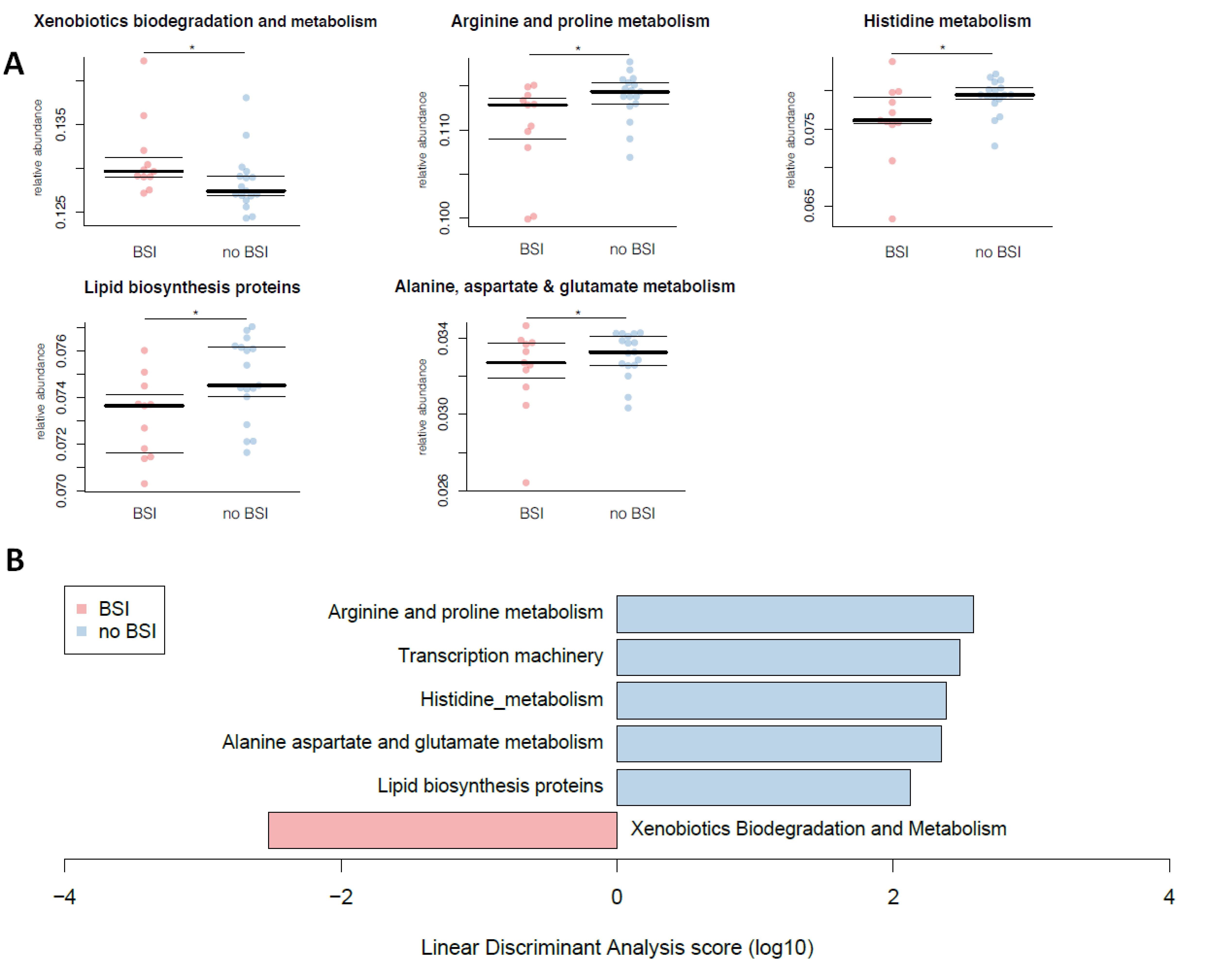
A
Xenobiotics biodegradation and metabolism
Arginine and proline metabolism
Histidine metabolism
Lipid biosynthesis proteins
Alanine, aspartate & glutamate metabolism
relative abundance
BSI
no BSI
0.125
0.135
0.100
0.110
0.065
0.075
0.070
0.072
0.074
0.076
0.026
0.030
0.034
*
B
BSI
no BSI
Arginine and proline metabolism
Transcription machinery
Histidine_metabolism
Alanine aspartate and glutamate metabolism
Lipid biosynthesis proteins
Xenobiotics Biodegradation and Metabolism
−4
−2
0
2
4
Linear Discriminant Analysis score (log10)
